# Supplementary material for: LTA4H extensively associates with mRNAs and lncRNAs indicative of its novel regulatory targets
Source: PeerJ. 2023 Mar 10;11:e14875. doi: 10.7717/peerj.14875 (PMC10010175; doi:10.7717/peerj.14875)
Supplement: File S2 [file peerj-11-14875-s005.zip › Raw data files/Reference_genome/Length_distribution_across_Genomic_Regions_Bar.pdf]

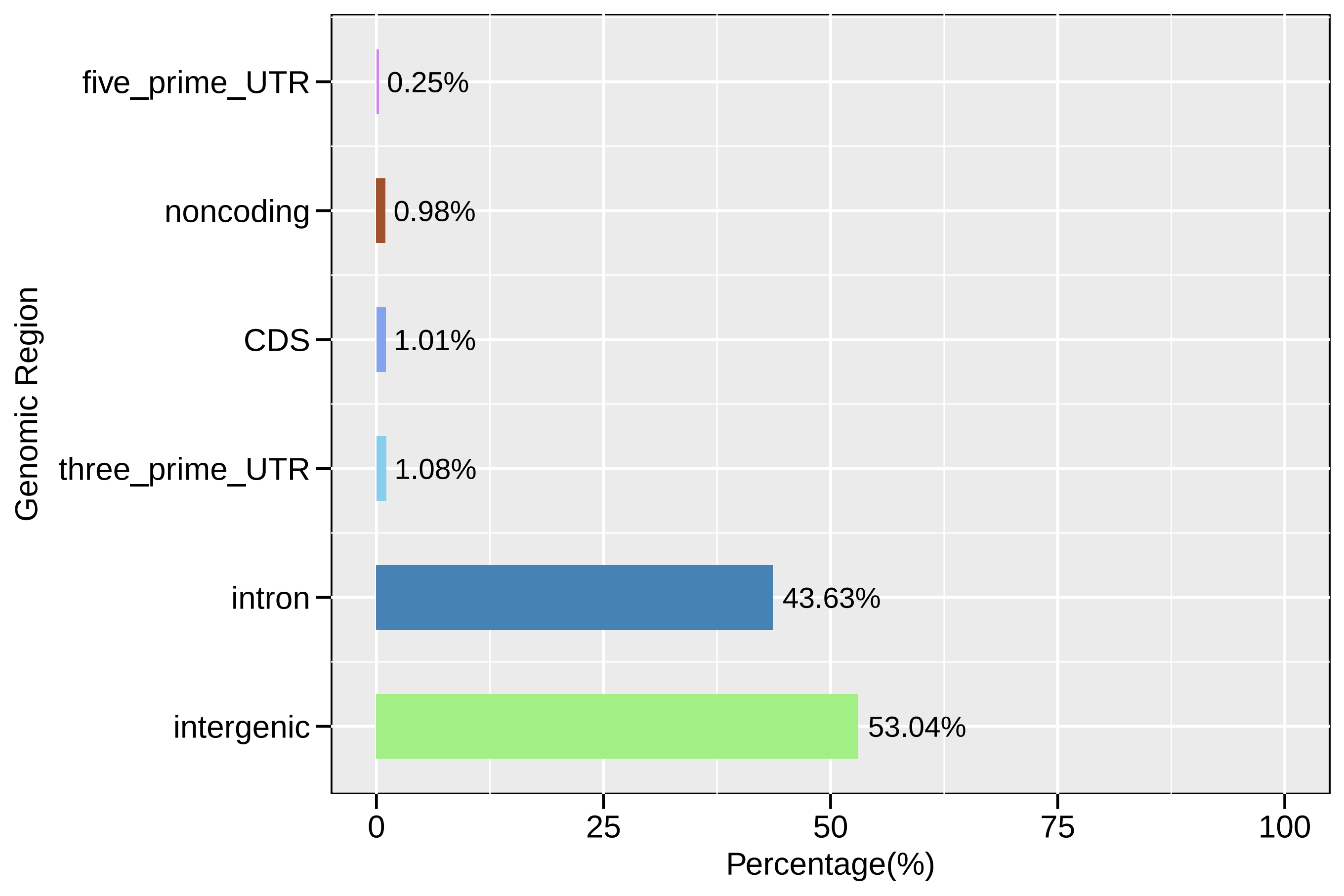
five_prime_UTR
noncoding
CDS
three_prime_UTR
intron
intergenic
0.25%
0.98%
1.01%
1.08%
43.63%
53.04%
Genomic Region
0
25
50
75
100
Percentage(%)
